# Supplementary material for: Field evaluation of DNA detection of human filarial and malaria parasites using mosquito excreta/feces
Source: PLoS Negl Trop Dis. 2020 Apr 8;14(4):e0008175. doi: 10.1371/journal.pntd.0008175 (PMC7170280; doi:10.1371/journal.pntd.0008175)
Supplement: S1 Table — (DOCX) [file pntd.0008175.s002.docx]

| **Community** | **Total mosquitoes** | **No. GA sl** | **% GA sl** | **No. FU sl** | **% FU sl** | **No. OTH An** | **% OTH An** | **No. All anophelines** | **% All anophelines** | **No. CU** | **% CU** | **No. OTH** | **% OTH** |
| --- | --- | --- | --- | --- | --- | --- | --- | --- | --- | --- | --- | --- | --- |
| Sekyerekura | 760 | 645 | 84.9 | 79 | 10.4 | 26 | 3.4 | 750 | 98.7 | 8 | 1.1 | 2 | 0.3 |
| Dugli | 1,571 | 1,386 | 88.2 | 168 | 10.7 | 9 | 0.6 | 1,563 | 99.5 | 8 | 0.5 | 0 | 0.0 |

GA sl= *An. gambiae* sensu lato; FU sl= *An. funestus* sensu lato; OTH An= other anophelines; CU= *Culex* sp; OTH= other genera (these included one *Mansonia* sp and one which was not identified due to a damaged carcass).
