# Supplementary material for: Field evaluation of DNA detection of human filarial and malaria parasites using mosquito excreta/feces
Source: PLoS Negl Trop Dis. 2020 Apr 8;14(4):e0008175. doi: 10.1371/journal.pntd.0008175 (PMC7170280; doi:10.1371/journal.pntd.0008175)
Supplement: S2 Table — (DOCX) [file pntd.0008175.s003.docx]

| **Method** | **No. GA sl (%)** | **No. FU sl (%)** | **No. OTH An (%)** | **No. CU (%)** | **No. OTH (%)** |
| --- | --- | --- | --- | --- | --- |
| Battery powered aspirator (indoors) | 2,005 (88.1) | 230 (10.1) | 32 (1.4) | 9 (0.4) | 0 |
| *Anopheles* gravid trap | 15 (83.3) | 2 (11.1) | 0 | 0 | 1 (5.6) |
| CDC Box gravid trap | 8 (27.6) | 12 (41.4) | 3 (10.3) | 6 (20.7) | 0 |
| BG-sentinel trap | 3 (37.5) | 3 (37.5) | 0 | 1 (12.5) | 1 (12.5) |

GA sl= *An. gambiae* sensu lato; FU sl= *An. funestus* sensu lato; OTH An= other anophelines; CU= *Culex* sp; OTH= other genera
